# Complete mitochondrial genome analysis of Japanese Spanish mackerel (*Scomberomorus niphonius*) with phylogenetic consideration

**DOI:** 10.1080/23802359.2017.1280700

**Published:** 2017-02-02

**Authors:** Yan Jin, Longling Ouyang, Hongxiang Zheng, Yu Lin, Menghan Zhang, Shengfa Li, Jiahua Cheng

**Affiliations:** a Key Laboratory of Marine and Estuarine Fisheries, Ministry of Agriculture of China, East China Sea Fisheries Research Institute, Chinese Academy of Fishery Sciences, Shanghai, China;; b MOE Key Laboratory of Contemporary Anthropology, School of Life Sciences, Fudan University, Shanghai, China

**Keywords:** *Scomberomorus niphonius*, mitochondrial genome, genome structure, gene order, evolutionary relationships

## Abstract

The complete mitochondrial DNA (mtDNA) of Japanese Spanish mackerel (*Scomberomorus niphonius*) was cloned and sequenced. The total length of the mitochondrial genome is 16,646 bp with an accession number KY228987. Thirty-seven genes are identified in total, including 13 protein-coding genes, 22 transfer RNA (tRNA) genes, 2 ribosomal RNA (rRNA) genes and a putative D-loop region. Among these genes, 9 are encoded on the light strand, while others are encoded on the heavy strand. The overall base composition of mitogenome is 28.39% for A, 16.14% for G, 26.52% for T, 28.96% for C, respectively, with a slight higher A + T content (54.91%). The phylogeny analysis based on 18 COI amino acid sequences suggested that *S*. *niphonius* and the other two species from *Scomberomorus* (Scombridae) formed a cluster apart from the one comprising other genus from Scombridae. The complete mitogenome may shed light on the future study of genetic mechanism of *Scomberomorus niphonius*.

Japanese Spanish mackerel (*Scomberomorus niphonius*) belongs to scombrid family (Scombridae), it is a commercially important species in the East China Sea, Yellow Sea, and Seto Inland Sea (Shui et al. 2009). For effective conservation of *S. niphonius* in the northwestern Pacific, it is essential to obtain the information of its genetic background. An adult fish of *S. niphonius* was collected in the gulf of Shanmen, China (29°00′N, 121°40′E). Its DNA was extracted and stored at −80 °C in East China Sea Fisheries Research Institute. The complete mitochondrial genome sequence of *S. niphonius* was analyzed and submitted to the GenBank database with an accession number KY228987.

The total length of this mitochondrial genome was 16,646 bp. Its overall base composition was 28.39% for A, 16.14% for G, 26.52% for T, 28.96% for C, with a slight higher A + T content (54.91%). This mitogenome contained 13 protein-coding genes, 22 transfer RNA (tRNA) genes, 2 ribosomal RNA (rRNA) genes, and a putative D-loop region. Among these genes, 28 were encoded by heavy strand, while 9 were encoded by light strand.

The overall base composition of the 13 protein-coding genes was 25.8% for A, 30.2% for C, 15.6% for G and 28.3% for T with a significant bias of cytosine. All the protein-coding genes contained the same start codon ATG except the gene cytochrome c oxidase subunit I (COI), which contained GTG instead. However, the termination codons of the 13 protein-coding genes were various, with either TAA, TAG, T––, AGA, or AGG. The incomplete termination codons, which were often found within the mitochondrial genomes of teleost fishes, were completed via posttranscriptional polyadenylation (Chu et al. 2013; Wang et al. 2016). The overlap between ATPase subunit 8- and ATPase subunit 6-coding gene was found in the mitogenome sequence of S. *niphonius*, which appears to be common in most vertebrate mitochondrial genomes, and its size in fishes (7–10 bp) smaller than that in mammals (40–46 bp). The length of 22 tRNA genes ranged from 67 to 74 bp. All tRNA genes could be folded into a typical cloverleaf structure except for tRNA-Ser (AGY), which lost the dihydrouridine arm and formed a simple loop with 12 unpaired nucleotides (Chu et al. 2013). The 12S and 16S rRNA genes were located between the tRNA-Phe and tRNA-Leu genes and were separated by the tRNA-Val gene with the same situation found in other vertebrates (Song et al. 2016; Zou et al. 2016). The control region of 959 bp was located between tRNA-Pro and tRNA-Phe genes.

The phylogenetic position of *S*. *niphonius* was reconstructed based on 18 COI amino acid sequences using the neighbour-joining (NJ) method. The phylogenetic tree showed that *S*. *niphonius* was in the clade of order Scombriformes (Figure 1). Species from family Scombridae, Nomeidae, Centrolophidaea, and Trichiuridae of Scombriformes were clustered into different sister groups (Figure 1). *S*. *niphonius* and the other two species from *Scomberomorus* (Scombridae) formed a cluster apart from the one comprising other genus from Scombridae in consistent with the study of Jondeung and Karinthanyakit (2010).

**Figure 1.**
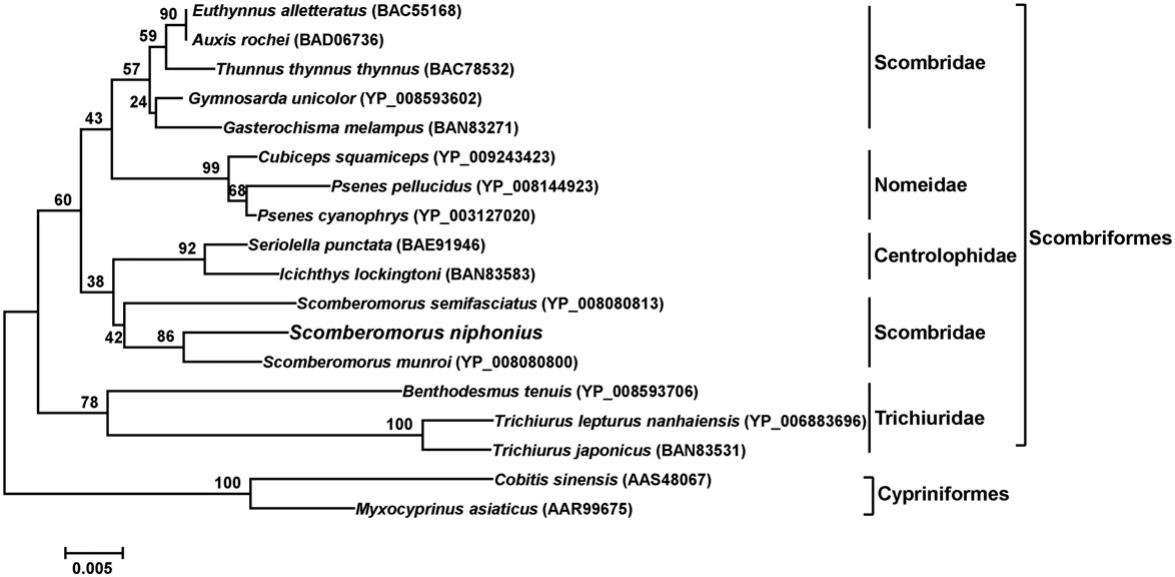
Phylogenetic position of *S. niphonius* derived from the NJ method of 18 COI amino acid sequences. Bootstrap analysis was based on 1000 re-samplings. All accession numbers are presented in the phylogeny tree. Two COI amino acid sequences from order Cypriniformes were chosen as an arbitrary outgroup.
